# Arousal influences olfactory abilities in adults with different degree of food neophobia

**DOI:** 10.1038/s41598-020-77428-w

**Published:** 2020-11-25

**Authors:** Leonardo Menghi, Iuliia Khomenko, Michele Pedrotti, Danny Cliceri, Eugenio Aprea, Isabella Endrizzi, Annachiara Cavazzana, Franco Biasioli, Davide Giacalone, Flavia Gasperi

**Affiliations:** 1 Center Agriculture Food Environment, University of Trento, Via E. Mach, 1, 38010 San Michele all’Adige, TN Italy; 2 Department of Technology and Innovation, University of Southern Denmark, Campusvej 55, 5230 Odense, Denmark; 3 Department of Food Quality and Nutrition, Research and Innovation Centre, Fondazione Edmund Mach, Via E. Mach, 1, 38010 San Michele all’Adige, TN Italy; 4 Department of Food Quality and Design, Wageningen University, P.O. Box 8129, 6700 EV Wageningen, The Netherlands; 5 Department of Otorhinolaryngology, Smell and Taste Clinic, Technische Universität Dresden, Fetscherstraße 74, 01307 Dresden, Germany

**Keywords:** Human behaviour, Feeding behaviour, Olfactory system, Mass spectrometry

## Abstract

Food neophobia, i.e., the aversion to novel foods, and olfaction are both factors strongly affecting food choices. Mounting evidence suggests a higher arousal towards food as a key factor underlying the reluctance to eat what is unfamiliar to us. As the role of olfaction behind this phenomenon is poorly understood, we explored the associations between food neophobia and trait anxiety, olfactory functions (odor threshold, discrimination and identification) and retronasal aroma release from a reference food in a healthy cohort of 83 adult volunteers. We grouped participants in Low-Neophobics or neophilics (n = 35), Medium-Neophobics (n = 32) and High-Neophobics (n = 16) according to the widely recognized Food Neophobia Scale. Participants with higher neophobic tendencies were found to have marginally higher trait anxiety levels than neophilics (*p* = 0.10). A lower global olfactory functioning and odor discrimination abilities characterized High-Neophobics, while Medium-Neophobics showed a higher odor sensitiveness than Low-Neophobics. Lastly, High-Neophobics showed a lower extent of retronasal aroma release, likely due to a shorter duration of oral processing and higher anxiety-related physiological responses (such as breathing rate). In summary, this study supports the assumption that the conflicting relationship that neophobics have with food may be led by higher levels of arousal toward foods, rather than different chemosensory functions.

## Introduction

Defined as the reluctance to eat unfamiliar foods^1^, Food Neophobia (FN) is a multidimensional phenomenon classified as part of the spectrum of feeding difficulties. An adaptive trait shared by all omnivores, FN sits at the core of the so-called “*omnivore’s dilemma*”^2,3^: humans are predisposed to exploit a vast and diverse set of nutritional resources to promote chances of survival but, at the same time, need to avoid ingestion of novel foods that might be potentially harmful^4^. FN is a developmentally appropriate behavior at an early age of an individual’s lifespan^1,5,6^, but it can persist into middle childhood and adulthood if tailored interventions are not provided^7^. Despite this, evidence of how FN may influence eating behaviors in adults, while growing e.g.^8–15^, is still relatively limited.

In adults, FN is likely to be associated to health-related issues such as higher body weight^14–16^ and overall reduced dietary quality and variety^9,16^. Interestingly, the latter association seems to extend beyond rejection of novel foods to encompass items that might be considered commonly consumed^8,9^. This suggests that FN may have a more pervasive influence on food preferences and intake, which is not limited to unfamiliar food. The heritability of FN is well-documented and the contribution of genetic factors on this feeding behavior seems to be still operating in adulthood (especially in women) with an estimated magnitude between 61 and 69%^11,17^. FN is considered to be a stable^18^ trait that co-varies with other factors like gender, age^12,19^, personality traits^5,11,20^, income or educational level^21^. However, the fear related to the unpleasantness of (novel) food’s sensory cues still remains a key determinant on its refusal^5^. This may be explained by the way neophobic individuals process sensory experiences as evidenced in several studies focusing on *taste* e.g.^8,14,15,19,20^ while, quite surprisingly, much less is known about the sense of *smell*, i.e. olfaction.

Olfaction has a prominent role in many domains of our life, from supporting us to process and to encode emotions through odors to social interactions^10,22^. Driving the adoption of behaviors in response to chemosensory stimuli in the environment^10^, olfaction has the important evolutionary function of alerting individuals from the ingestion of potentially aversive substances and recognizing foods useful for survival^23^. Moreover, olfaction is extremely influential on food choices and preferences as it is a major contributor to food flavor^24^, which is crucial when it comes to the sensory evaluation and appreciation of food.

Neophobic individuals are reportedly different from “neophilics” (i.e. individuals willing to try and accept novel foods) in the way they explore the olfactory environment (see^10^ for a review). Raudenbush et al*.*^13^ reported that adults less willing to try novel foods rated a series of odors (both familiar and unfamiliar) as less pleasant and less intense, and tended to use smaller sniff magnitudes compared to neophilics, as measured in an odor detection task. These findings suggested an attempt made by neophobics to avoid any potential odor-related experience with foods^25^. Moreover, neophobics are reportedly less accurate at naming odors, possibly reflecting a passive attitude to explore the chemosensory environment^26^. Finally, a recent study measured the spontaneous exploratory behavior that toddlers had towards pleasantly and unpleasantly odorized bottles focusing on mouthing behaviors and reported a positive association in boys between smell reactivity and FN^27^. Similarly, in a study conducted by Farrow and Coulthard^28^ involving toddlers aged 5–10, parents’ perceptions of their children’s taste/smell sensitivity was associated with higher levels of FN and anxiety. Interestingly, the results revealed that children's sensory sensitivity (i.e. individual differences in the detection of, and reaction to, chemosensory stimuli) mediated the relationship between anxiety and children’s tendency to selective feeding behavior. These results suggest that a higher sensitivity to chemosensory stimuli may explain why more anxious children are more likely to be selective eaters.

Whether results from these studies would replicate in an adult population is unknown, although it is worth noting that a positive relationship between trait anxiety and FN has also been observed in adults in at least one study^1^, where neophobics showed higher arousal responses (i.e. increased heart rate, galvanic skin responses and respiration) compared to neophilics when presented with pictures of food stimuli^29,30^. Similar insights come from large-scale studies, which reported that differences in liking and sensory responses toward food items eliciting *“warning”* (e.g., bitter, sour, astringent)^8^ or pungent^20^ sensations were not affected by differences in taste sensitivity, operationalized in terms of PROP (6-n-propylthiouracil) responsiveness and/or fungiform papillae density.

Taken collectively, the existing research would suggest that the feeding behavior of adult neophobics is more likely driven by a global higher arousal responsiveness toward foods, rather than by a higher chemosensory functioning. Since neophobics tend to be more alert when confronted with food^30^, whether familiar or unfamiliar^8,9^, we hypothesized that this behaviour may also be explained by the different way in which neophobics react to the stimulation of their olfactory epithelium from food odorants, which can occur when odorants are inhalated through the nostrils (orthonasal olfaction) or released in the mouth during consumption and then exhalated via the back of the throat (retronasal olfaction).

Situated within this context, the present study aimed at identifying differences in olfactory abilities from adult individuals with different selective/restrictive attitudes towards food, and to infer a possible effect of arousal underlying these differences.

To the best of our knowledge, no studies reporting the associations between olfactory functions, arousal responses and FN have been carried out yet. To address this issue, we studied a healthy cohort of adults assessed for orthonasal functioning through the Sniffin’ Sticks battery^31^. For each subject, we monitored *in real time* for retronasal aroma release during the consumption of a reference food product (a strawberry flavored candy) through *nose-space analysis* (NS), the analysis of volatile compounds concentration in the nose, with Selected-Ion Flow-Tube Mass Spectrometry (SIFT-MS)^32^. Participants also completed an online socio-demographic questionnaire and the widely used Food Neophobia Scale (FNS)^1,8^ to investigate neophobic traits. Lastly, the trait anxiety subscale of the State-Trait Anxiety Inventory Questionnaire (STAI-T)^33,34^ was used to examine the hypothesis that neophobics’ responses to olfactory cues may be led by an overall higher arousal state mediated by anxiety rather than by a higher sensitivity to odors.

## Results

### Cohort description

Overall, we collected data from 83 volunteers of which 48 were females (57.8%, mean age = 41.4; SD = 11.9) and 35 were males (42.2%, mean age = 42.2; SD = 11.4). The mean age of participants was 41.7 ± 11.7 years old. A higher proportion of females were normal-weight, while males were more likely to be overweight or obese (*χ*^2^ = 9.98; *p* = 0.006).

The mean FN level in the sample was 24.8 ± 11.5, in line with existing data on the Italian population (27.4 ± 11.7)^8^ and a mean level of anxiety proneness of 39.9 ± 9.3, indicative of moderate anxiety (38–44)^34^. We did not observe significant correlations between age and FN (R = 0.16, *p* = 0.14), unlike trait anxiety where a negative correlation was detected (R = −0.28, *p* = 0.009). BMI (Body Mass Index) was slightly associated with FN (R = 0.19, *p* = 0.09) but not with STAI-T scores (R = −0.14, *p* = 0.19). Finally, no gender differences in either FN (*t* = 1.264, *p* = 0.21) and STAI-T (*t* = 1.14, *p* = 0.25) were observed.

With regard to the olfactory data, we identified 76 normosmic and 7 hyposmic individuals. Their averaged global olfactory abilities, in terms of TDI (odor Threshold, Discrimination, Identification), were mostly higher than the 50th percentiles of the European population^35^, thus offering a satisfactory olfactory performance (see Supplementary Table S1). In line with previous reports^35–38^, we found considerable negative associations between TDI, age and BMI, but no association between TDI and gender. Similar insights came from correlations between olfactory subtest scores (OT = Odor Threshold, OD = Odor Discrimination, OI = Odor Identification), age and BMI where OT was inversely correlated with BMI, while OD was inversely correlated with both age and BMI. No correlations were found for the OI task and all the olfactory subtests for gender.

Lastly, according to the extent of retronasal aroma release (area under the curve: AUC), results revealed no associations with age and BMI or differences in terms of gender for each monitored compound.

The reader is further referred to Supplementary Materials where significant correlations between olfactory performance scores, retronasal aroma release, gender, age and BMI are displayed (Figs. S1, S2 and Table S2).

### Participants’ segmentation and characterization according to their food neophobia level

Participants were grouped according to their FN level using the *cut-offs* proposed by Laureati et al*.*^8^ based on a large Italian sample. The group with neophilic tendencies (Low-Neophobics: LN) fell into the first quartile of the FNS scores distribution (FNS score ≤ 18) and represented the 42.2% of the total sample (48.5% female; mean FNS score: 14.8). The group with medium neophobic tendencies (Medium-Neophobics: MN) included participants that reported FNS scores between 18 and 36 and represented the 38.6% of the sample (62.5% female; mean FNS score: 26.3). Lastly, the group with highest neophobic traits (High-Neophobics: HN) fell into the highest quartile of the FNS scores distribution (FNS score ≥ 36) and accounted the 19.2% of participants (HN; 68.7% female—mean FNS score: 43.8). The three groups did not differ for age (F = 1.03; *p* = 0.36), BMI (F = 0.81; *p* = 0.44) or gender proportions (*χ*^2^ = 2.29, *p* = 0.31).

### Associations between trait anxiety and food neophobia

STAI-T scores were submitted to a 1-*way* ANOVA to assess possible differences in anxiety proneness between FN groups. Results revealed a trend (F = 2.32; *p* = 0.10) by which higher FN levels were associated to a higher anxiety proneness, further corroborated by a weak positive correlation between the two variables (R = 0.14; *p* = 0.20). Although substantial differences between FN groups were not observed, higher neophobic traits showed averaged values of STAI-T scores (MN: 42.0; HN: 41.31) commonly indicative of moderate levels of anxiety compared to the LN group, which showed a lower mean anxiety proneness (37.4)^34^.

### Associations between olfactory performances and food neophobia

**Figure 1:**
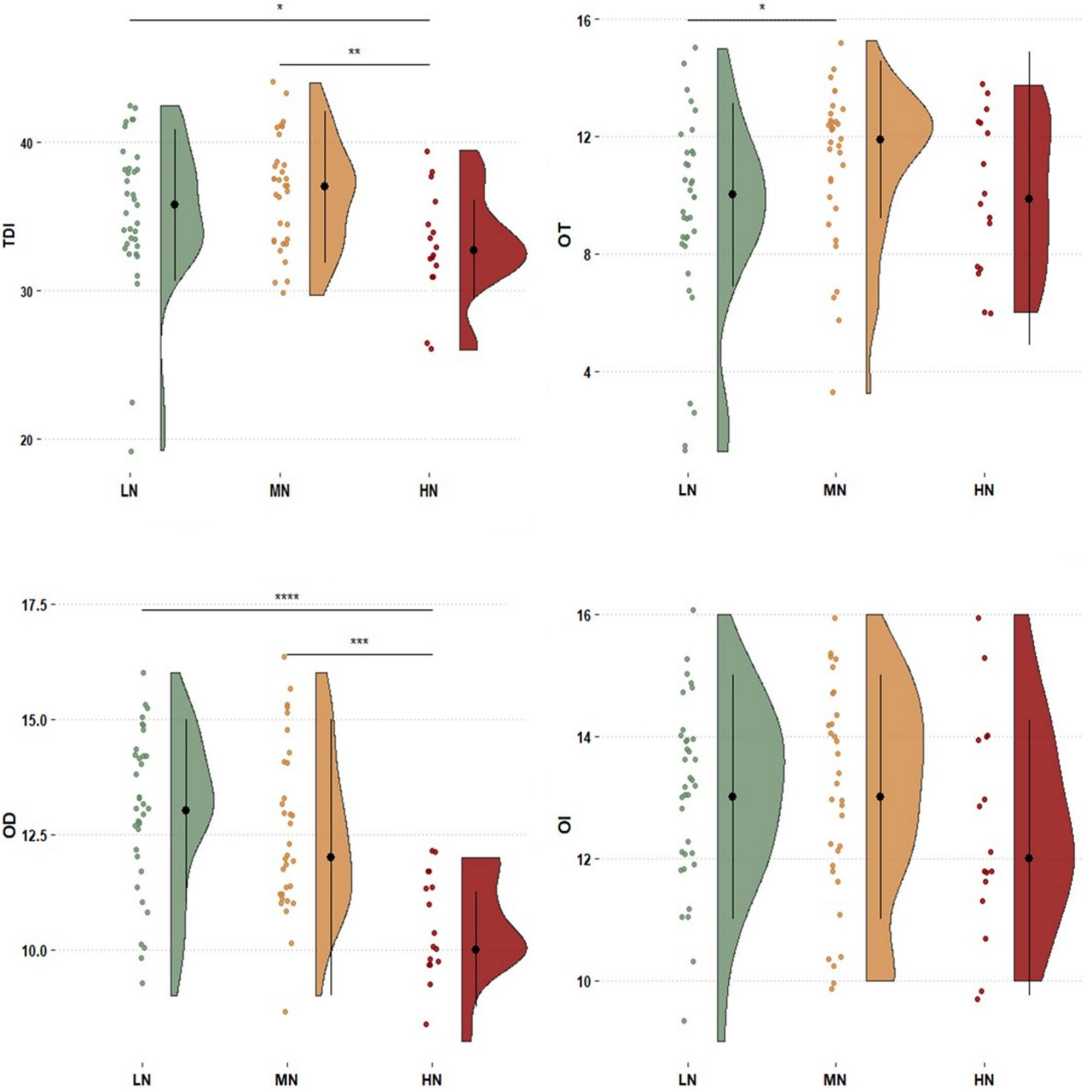
Raincloud plot showing the differences on both global (TDI) and relative subtests (OT: Odor Threshold; OI: Odor Identification, OD: Odor Discrimination) olfactory assessments as a function of FN level (LN: Low-Neophobics; MN: Medium-Neophobics; HN: High-Neophobics). The plots provide a representation of data distribution (the ‘*cloud’*), individual raw observations (the ‘*rain*’), the median (black filled circle) ± IQR (perpendicular) within each FN level for both global and subtests olfactory performance. Only statistically significant pairwise differences observed after post hoc Dunn’s test with Bonferroni correction are presented (*p < 0.05; **p < 0.01; ***p < 0.001; ****p < 0.0001).

To explore whether FN groups dealt differently with olfactory cues, differences on both olfactory subtests (OT, OD, OI) and TDI between FN levels were assessed through separate Kruskall–Wallis tests. Overall, a main effect of neophobic traits on TDI scores was observed (H = 7.51, *p* = 0.02), with HN reporting considerably worse global olfactory performances compared to LN and MN. A main effect of FN on OT was also detected (H = 5.46, *p* = 0.03) with LN showing higher olfactory thresholds (i.e., lower sensitivity) compared to MN (Dunn’s test). In this task, no differences were reported for HN when compared to LN or MN. We found strong differences in the OD task (H = 21.08; *p* < 0.001), which indicated that both LN and MN outperformed HN. Conversely, we did not find differences in OI abilities between FN levels (H = 1.16; *p* = 0.56). Raw scores, distributions and significant pairwise comparisons between FN levels for both TDI and olfactory subtests are displayed in Fig. 1.

### Associations between retronasal aroma release and food neophobia

#### Multivariate exploration

**Figure 2:**
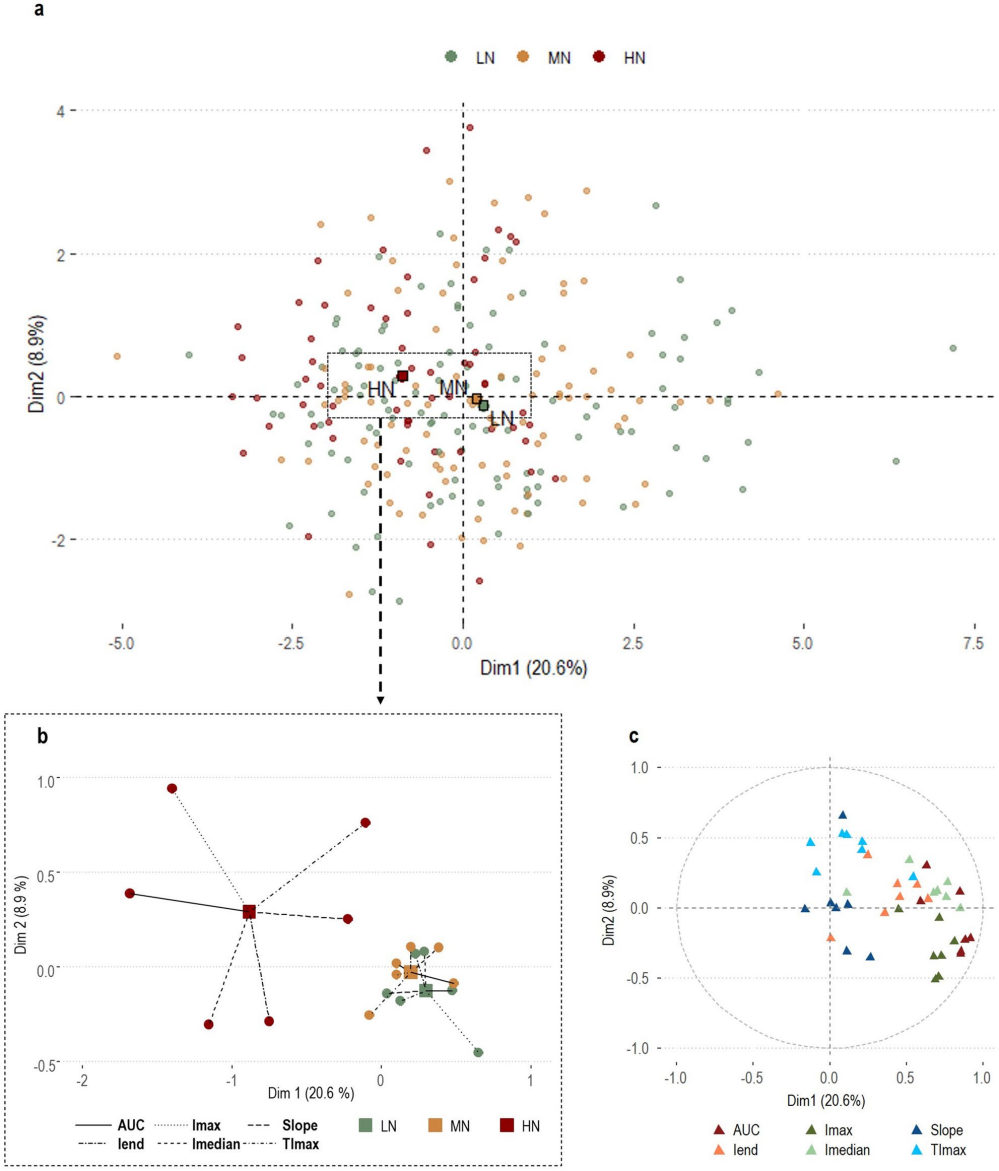
First two factors of the individual factor map (**a**) from the MFA based on matrices of SIFT-MS parameters. Large squares (light green square, light orange square, brown square) are MFA centroids while the circles (light green circle, light orange circle, brown circle) represent participants’ position in the bi-dimensional space colored according to their FN level (LN, MN, HN). Each MFA centroid is associated (**b**) with partial MFA groups points for the six SIFT-MS parameters (small circles with same colour as the corresponding MFA centroid). (**c**) Variable factor map from the MFA model. Dots represent correlations between SIFT-MS parameters (for the 7 monitored compounds) and the first two significant dimensions of the MFA model.

To explore overall differences in retronasal aroma release between FN levels, data from the 7 monitored compounds grouped according to the SIFT-MS extracted parameters (AUC, I_max_, I_median_, I_end_, TI_max_, Slope) were submitted to a Multiple Factor Analysis (MFA, Fig. 2). Amount of acetone (endogenous compound) was not considered in this analysis.

Participants were uniformly distributed over the first two dimensions of the MFA score plot, together accounting for 29.5% of variance (Fig. 2a). Dim.1 (20.6% of variance) was positively associated with a higher proportion of replicates from LN and MN while most replicates from HN were located on the opposite end of the plot, suggesting a contrasting behaviour. Dim.2 (8.9% of variance), further separated FN levels as most of replicates from HN were positively related to this dimension, whereas LN and MN were not clearly separated over Dim.2. Results for additional MFA dimensions (Dim.3 = 8.3%; Dim. 4 = 5.6% of variance) are given in the Supplementary Material (Figs. S3, S4). Overall, the first four dimensions of the model accounted for 43.4% of the total variance.

A deeper characterization of the FN groups can be appreciated in Fig. 2b, where MFA centroids (LN, MN, HN) and related partial MFA points (one of for each of the six groups of parameters considered) are visible. Starting from MFA centroids, each partial point projects the position of that FN level according to the group of variables considered^39^. We observed the largest differences between FN levels in terms of time-independent parameters (AUC, I_max_, I_median_) with LN and MN showing positive partial projections in Dim.1 (which accounted for the most of variation) unlike HN, which showed a contrasting behavior. Associations with Dim. 2, instead, were less helpful to explain differences between FN levels as mostly related to the origin of the MFA model which is indicative of a lower explanatory power. Overall, these results suggest that HN showed a lower extent of retronasal aroma release than both LN and MN not mediated by different release kinetic patterns. Partial projections of SIFT-MS extracted parameters within FN levels can be further interpreted by taking into account the variable factor map of the MFA model (Fig. 2c). Dim. 1 well represents both the increasing direction and the correlations between all time-independent parameters (i.e. AUC, I_max_, I_median,_ I_end_). Since both these parameters appear grouped together and are positively correlated to the first dimension, a twofold consideration can be drawn. Firstly, as most of variables within groups were strongly correlated and far from the origin, these groups were well represented by the MFA model^39^. Secondly, the positive correlation with the first dimension of the model gave information on the increasing direction of these variables and confirmed that individuals in the LN and MN groups, with positive coordinates for Dim.1, showed a higher extent of retronasal aroma release compared to the HN group, located on the opposite side of this dimension.

By contrast, the second dimension represented well only the TI_max_ group with most of variables being grouped together and far from the origin. In other words, individuals with positive coordinates on Dim. 2 reached the maximum intensity of most of VOCs later than the ones with negative coordinates on this dimension. Lastly, most variables related to Slope were close to the origin of axes, meaning that they were not well represented by the MFA model and, therefore, less helpful in explaining variance between FN levels.

Overall, results from MFA suggested that HN was associated to a lower extent of retronasal aroma release. Additionally, no clear indication on different release patterns between the compounds monitored were observed, suggesting similar kinetics within individuals.

#### Univariate approach

**Figure 3:**
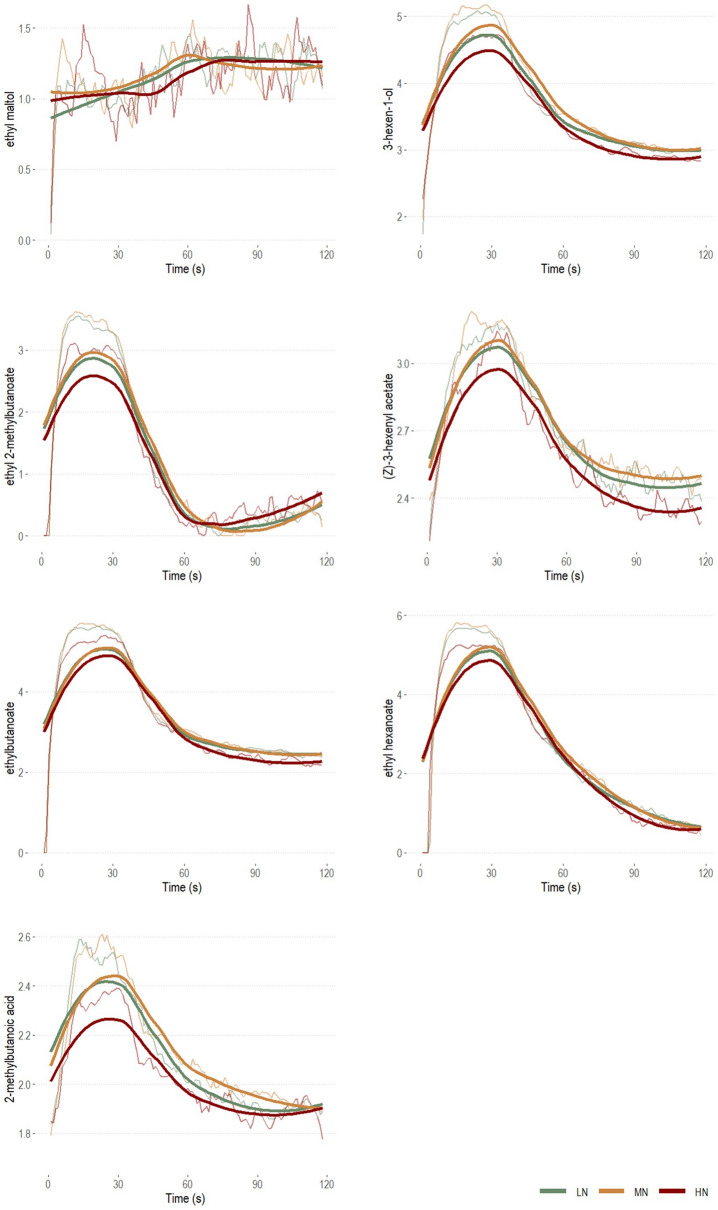
Log-transformed median (transparent) and smoothed (bold) release curves for the 7 monitored VOCs by SIFT-MS as a function of FN level.

The data on retronasal aroma release were further explored univariately to further assess significant differences between FN levels. Table 1 reports all VOCs and T-I curves related parameters coupled with *p* values from Kruskall Wallis tests, as well as pairwise comparisons according to Dunn’s test with the Bonferroni adjustment. Figure 3 displays FN levels’ median and smoothed curves for each compound.

**Table 1:** VOCs monitored by SIFT-MS and related T-I curves related parameters.

Sum formula	VOCs	Parameter	LN	MN	HN	H	*p* value
C_7_H_8_O_3_	Ethyl maltol	*AUC*	3.25 ± 0.42^ab^	3.35 ± 0.44^b^	3.18 ± 0.39^a^	6.34	**0.04**
		*I*_*max*_	1.24 ± 0.31	1.21 ± 0.34	1.19 ± 0.6	0.94	0.63
		*I*_*median*_	0 ± 0	0 ± 0	0 ± 0	0.87	0.65
		*I*_*end*_	0 ± 1.09	0 ± 1.03	0 ± 1.05	0.47	0.79
		*TI*_*max*_	29 ± 40.5	30 ± 44	38.5 ± 44.25	4.02	0.13
		*Slope*	0 ± 0	0 ± 0	0 ± 0	0.02	1
C_6_H_12_O	3-Hexen-1-ol	*AUC*	8.86 ± 0.68^b^	8.91 ± 0.54^b^	8.61 ± 0.44^a^	20.46	**< 0.001**
		*I*_*max*_	6.05 ± 0.7^b^	6.04 ± 0.56^b^	5.84 ± 0.65^a^	14.21	**< 0.001**
		*I*_*median*_	3.49 ± 0.5^b^	3.56 ± 0.45^b^	3.33 ± 0.35^a^	15.25	**< 0.001**
		*I*_*end*_	2.99 ± 0.56^b^	2.99 ± 0.38^b^	2.89 ± 0.38^a^	7.01	**0.03**
		*TI*_*max*_	17 ± 15	18 ± 14	20 ± 13.5	1.33	0.51
		*Slope*	0.13 ± 0.1	0.11 ± 0.09	0.12 ± 0.1	3.73	0.16
C_7_H_14_O_2_	Ethyl 2-methyl butanoate	*AUC*	7.01 ± 0.83^b^	7.13 ± 0.74^b^	6.65 ± 0.88^a^	20.10	**< 0.001**
		*I*_*max*_	4.8 ± 0.66^b^	4.93 ± 0.78^b^	4.54 ± 0.9^a^	16.39	**< 0.001**
		*I*_*median*_	0.84 ± 0.97^b^	0.91 ± 0.99^b^	0 ± 0.9^a^	6.72	**0.03**
		*I*_*end*_	0 ± 0.46	0 ± 0.64	0 ± 0.63	0.16	0.92
		*TI*_*max*_	13 ± 12.5	12 ± 15	13 ± 12.75	0.98	0.61
		*Slope*	0.38 ± 0.69	0.34 ± 0.7	0 ± 0.56	5.02	0.08
C_8_H_14_O_2_	(*Z*)-3-hexenyl acetate	*AUC*	7.5 ± 0.45	7.56 ± 0.56	7.38 ± 0.56	5.19	0.07
		*I*_*max*_	4.27 ± 0.46^b^	4.3 ± 0.54^b^	4.16 ± 0.4^a^	8.82	**0.01**
		*I*_*median*_	2.66 ± 0.71	2.68 ± 0.88	2.66 ± 0.74	1.16	0.56
		*I*_*end*_	2.41 ± 0.77	2.46 ± 0.83	2.41 ± 0.85	0.53	0.77
		*TI*_*max*_	20 ± 19.5^b^	24 ± 22^a^	27 ± 35^a^	11.26	**< 0.001**
		*Slope*	0 ± 0.03	0 ± 0.09	0 ± 0.04	4.70	0.10
C_6_H_12_O_2_	Ethyl butanoate	*AUC*	9.15 ± 0.73^b^	9.2 ± 0.53^b^	8.94 ± 0.48^a^	18.24	**< 0.001**
		*I*_*max*_	6.92 ± 0.84^b^	6.86 ± 0.7^b^	6.55 ± 0.72^a^	19.67	**< 0.001**
		*I*_*median*_	3.02 ± 0.46^b^	3.06 ± 0.45^b^	2.92 ± 0.33^a^	9.34	**0.01**
		*I*_*end*_	2.38 ± 0.79	2.49 ± 0.68	2.28 ± 1.05	5.45	0.07
		*TI*_*max*_	0.19 ± 0.14	0.14 ± 0.14	0.16 ± 0.14	1.30	0.52
		*Slope*	16 ± 12.5	15 ± 15	17.5 ± 15.5	4.05	0.13
C_8_H_16_O_2_	Ethyl hexanoate	*AUC*	9.11 ± 0.82^b^	9.26 ± 0.69^b^	8.84 ± 0.74^a^	19.28	**< 0.001**
		*I*_*max*_	6.77 ± 0.82^b^	6.81 ± 0.65^b^	6.49 ± 0.88^a^	14.53	**< 0.001**
		*I*_*median*_	2.35 ± 0.91	2.46 ± 0.95	2.21 ± 1.18	1.34	0.51
		*I*_*end*_	0.75 ± 1.43	0.6 ± 1.3	0.48 ± 1.02	1.05	0.59
		*TI*_*max*_	14 ± 13	14 ± 13	16 ± 14.75	1.49	0.47
		*Slope*	0.21 ± 0.47^b^	0.14 ± 0.22^a^	0.21 ± 0.33^ab^	7.58	**0.02**
C_5_H_10_O_2_	2-Methylbutanoic acid	*AUC*	6.86 ± 0.37^b^	6.95 ± 0.42^b^	6.82 ± 0.25^a^	11	**< 0.001**
		*I*_*max*_	3.46 ± 0.51^b^	3.52 ± 0.5^b^	3.27 ± 0.46^a^	17.37	**< 0.001**
		*I*_*median*_	2 ± 0.34	2.07 ± 0.34	2 ± 0.24	5.82	0.05
		*I*_*end*_	1.91 ± 0.47	1.94 ± 0.47	1.9 ± 0.41	0.58	0.75
		*TI*_*max*_	18 ± 14.5	15 ± 17	16.5 ± 15.5	0.03	0.99
		*Slope*	0 ± 0.14	0 ± 0.12	0 ± 0.06	2.99	0.22

Median ± IQR are reported for each compound as a function of FN level (LN, MN, HN). Within each parameter, data are presented as log-transformed with the exception of TI_max_. *p* values were obtained through separate Kruskall Wallis tests, with significant ones highlighted in bold. Median ± IQR marked with different superscript letters by row indicate statistically significant differences (*p* < 0.05) according to post hoc Dunn’s test with Bonferroni adjustment.

Overall, results corroborated the ones from the MFA model (see Fig. 2). Amongst the six extracted parameters, the ones that showed most of variation between FN levels were those describing aroma release in a time-independent fashion. Accordingly, considerable differences were found in the overall amount of VOCs released (AUC; except for (Z)-3-hexenyl acetate that showed a trend: *p* = 0.07) and in the maximum intensity released (I_max_, except for ethyl maltol). Fewer differences were observed for the median intensity (for 4 compounds out of 7 with 2-methylbutanoic acid showing a trend: *p* = 0.05) and the averaged released intensity of the last five seconds of the NS task (I_end_; only for 3-hexen-1-ol). The aforementioned differences always revealed LN and MN outperforming HN in terms of overall retronasal aroma release (Fig. 3). Conversely, differences in time-related parameters (i.e. Slope, TImax) were lower and compound independent. Slope showed differences only for ethyl hexanoate with LN showing a faster decrease in the exhalation of this VOC compared to MN. Only (Z)-3-hexenyl acetate showed differences for TI_max_, with LN reaching the maximum intensity released faster than the other two groups.

#### Association between food neophobia, retronasal aroma release and behavioural parameters

To corroborate the idea that neophobics may be led by a higher global arousal in their approach to foods, two different parameters were extracted from the NS task: the duration of oral processing and the breathing rate of participants within each replicate. Firstly, we found significant differences on duration of oral processing (i.e. the difference in terms of timing when participants put the sample in-mouth and the time they swallowed the candy) as a function of FN level (H = 11.93; *p* < 0.0001), with LN (Z = 2.38; *p* = 0.008) and MN (Z = 3.45; *p* = 0.003) chewing the sample for a longer time than HN.

**Figure 4:**
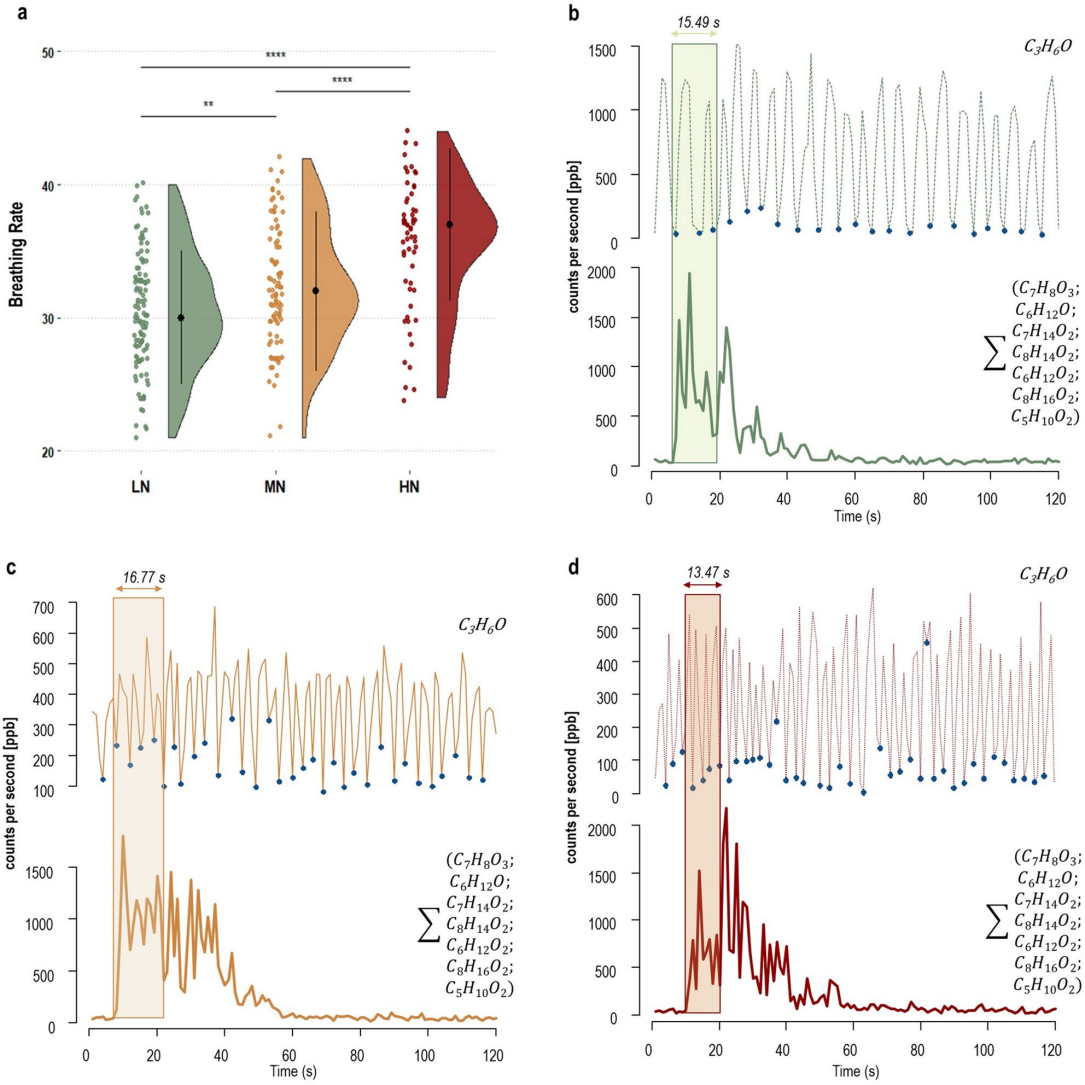
Raincloud plot (**a**) showing the differences in breathing rates between FN levels (LN: Low-Neophobics; MN: Medium-Neophobics; HN: High-Neophobics). The plot provides a representation of data distribution (the ‘*cloud’*), individual raw observations (the ‘*rain*’), the median (black filled circle) ± IQR (perpendicular) within each FN level. Only statistically significant pairwise differences observed after post hoc Dunn’s test with Bonferroni correction are presented (*p < 0.05; **p < 0.01; ***p < 0.001; ****p < 0.0001). **b**–**d:** examples of breathing behaviors from a replicate of three participants with similar biological characteristics (**b**: Female; 51 yo; BMI = 19.27; **c**: Female; 53 yo; BMI = 25.46; **d**: Female; 53 yo; BMI = 21.82) but different according to their FN level. The top trace shows the curve of acetone (C_3_H_6_O) within the task with relative local minima (blue filled circle). The bottom trace is the sum of the 7 monitored VOCs, which represents the volatilome of the candy. Lastly, the transparent rectangles report the duration of the oral processing (s) for each replicate considered.

Secondly, we estimated the breathing rate of participants for each replicate by counting the local minima sites from the acetone curves within the task. Results revealed a strong significant difference between the groups (H = 38.68; *p* < 0.0001), as HN were found to breathe faster than both MN (Z = 3.92; *p* < 0.0001) and LN (Z = 6.21; *p* < 0.0001). Moreover, a statistically significant difference was also observed between LN and MN, with LN undergoing the task with longer breathing cycles (Z = 2.49; *p* = 0.006). Pairwise comparisons according to post hoc Dunn’s test with Bonferroni correction between groups (panel a) and examples of breathing behaviors (panels b, c, d) between FN levels are displayed in Fig. 4.

## Discussion

This study assessed the contribution of olfaction (both orthonasal and retronasal) on food neophobia. We hypothesized that this avoidant/selective feeding behaviour may be linked to an adaptive usage of olfaction mediated by a higher arousal responsiveness towards foods.

In our adult cohort, approximately a fifth of individuals showed high neophobic tendencies (FNS ≥ 36)^8^, consistent with known distributions for the Italian population (26.2%; Laureati et al*.*^8^). Surprisingly, we did not find differences for age, gender or BMI between FN levels, as previously documented^8,12,16,19–21,38^, which is probably due to the smaller size of our cohort than the ones used by the aforementioned studies. However, this finding is advantageous in the present study as it allowed us to draw conclusions on the effects that FN may have on olfactory performances without possible confounders.

The idea that neophobics would respond differently to olfactory stimulation was confirmed by the olfactory assessment, as the HN group offered worse global olfactory performances than LN and MN.

Firstly, neophobics differ from neophilics by the way they explore the chemosensory environment. We found strong differences in the OD task between FN levels, with HN performing worse than MN and LN. Our findings are in line with a previous investigation, which assessed olfactory functioning on individuals showing other selective/restrictive feeding behaviors as anorexia nervosa^40^, and reported that anorectic patients offered poorer odor discrimination performances compared to a control group.

Secondly, participants with medium levels of FN showed a higher odor sensitivity than neophilics. These results seem consistent with Monnery-Patris et al*.*^27^, which explored smell and taste reactivity in toddlers and found a modestly higher smell reactivity among young neophobic boys.

A higher sensitivity to chemosensory stimuli has been reported to mediate the well-known relationship between FN and anxiety^1,28^. Also in this study, individuals with higher neophobic tendencies showed a higher anxiety proneness, albeit less strongly (*p* = 0.10) than previously documented^1^. However, according to^34^, our neophobic population (MN and HN) showed a mean level of anxiety commonly classified as higher compared to the one observed for neophilics, thus corroborating this hypothesis.

We found some unexpected results from the OI task. Assuming that odor identification is mediated by the degree of exposure one person has with the olfactory world^10^, we expected that milder levels of FN would outperformed HN in this task, as previously reported by Demattè et al*.*^26^. In that study, 167 individuals were asked to identify a series of 36 common odors presented in glass vials from a 90-label list (arranged in odor categories), which resulted in neophobic participants performing significantly worse in naming odors than neophilics. In contrast, our findings did not support differences between FN levels in the OI task. This unexpected result might be related to the task being easier than the one proposed by^26^, where participants correctly identified on average 38% of the odors provided. In the OI subtest, participants were asked to name highly common odors^31^ supported by a four-alternative forced-choice visual-worded paradigm. Cross-modal integrations between vision and olfaction in presence of congruent pictures^41^ or relevant sematic information^42^ have been proposed to magnify odor identification. Thus, it is possible that the presence of visual-worded cues, combined with the routine clinical purposes for which the Sniffin’ Sticks battery was designed^31^, made the task less apt to detect differences between FN levels.

Overall, our findings support the idea that a lower willingness to explore the chemosensory world does not have direct relevance once sensory perception of food occurs but, rather, that it would have led neophobics to develop an anxiety-mediated adaptive behaviour^43^ that protect them from exaggerated sensations of possible aversive outcomes associated with food items^1,25,28^.

The retronasal assessment supported the same interpretation. NS has been widely applied to study the complex network of factors affecting inter-individual differences on retronasal aroma release, such as oral processing^44,45^, physiological parameters^45,46^, age^47^, gender and ethnicity^46^ and BMI^47,48^. To the best of our knowledge, our study is the first in which a direct injection mass spectrometry technique was applied to get insights on behavioral traits underlying individuals with different attitudes towards food, thus opening new scenarios for further investigations of eating behaviors.

Results from the MFA model revealed that LN and MN showed an overall higher retronasal aroma release compared to HN. These findings were later confirmed by the univariate approach showing lower time-independent parameters (AUC, I_max_, I_median_, I_end_) in individuals with highest neophobic tendencies. By contrast, we did not find clear differences in terms of time-dependent parameters (Slope, TI_max_), suggesting that all compounds monitored had similar kinetics within individuals.

These findings were further corroborated by the shorter duration of oral processing showed by high FN participants, which is consistent with the existing literature. Labouré et al*.*^45^ reported that individuals exhaling the highest amount of aroma from a model solid food (i.e., a model cheese) showed the highest chewing activity, while Ruijschop et al*.*^48^ reported that a longer duration of oral processing tended to result in a higher retronasal aroma release during consumption of a fixed amount, as also found in our study.

It was therefore not surprising that individuals with higher neophobic tendencies underwent the task by breathing in a faster fashion. This is consistent with the previously reported increase in physiological arousal (in terms of pulse rate, Galvanic skin response and respiration rate) when neophobics are presented with food stimuli^29^. Breathing evaluation is a useful physiological marker of level of anxiety^49^, with anxious individuals showing larger respiratory rates in presence of anticipatory anxiety-inducing stimuli^50^. According to a previously proposed theoretical model on anxiety^51^, more anxious individuals would address stimuli with an increased arousal response to the potential of negative consequences, which manifests itself in enhanced anterior insular cortex processing. The same theoretical could be used to explain the way neophobics deal with chemosensory stimulations. Recently, Spinelli et al*.*^20^ presented data suggesting that liking and sensory perception of spicy (a “*warning*” sensation) foods is influenced by an increased arousal state associated with neophobic traits, which would modulate sensory and hedonic responses of individuals. Interestingly, this condition seem to be extended beyond the concept of familiarity^8,9^. Our findings supported this conclusion, as they were obtained using a common food item.

However, results have to be taken cautiously. The artificial condition in which individuals underwent the NS task (i.e., the unfamiliar laboratory environment or the oxygen cannula fitted in their nostrils) may have independently affected participants’ arousal state. This in turn may have influenced their behavior during the task inducing larger discrepancies between FN levels.

Moreover, our study did not provide a hedonic and a perception measurement of the candy. However, it can be reasonably assumed that our sample was in the acceptable range, as it was chosen to elicit a sensory perception (sweetness) which is well accepted as it is commonly related to energy dense foods.

Another potential limitation of the study is that the three levels of FN were defined on arbitrary *cut-offs* based on the FNS frequency distribution. While commonplace in the literature, e.g.^8,9,21^, a potential drawback of this approach is that it could downplay the influence of extremely neophobic individuals, given the well-known reluctance of such individuals to participate in similar studies. In future studies, it would be interesting to see whether the magnitude of effects observed between FN levels, for example in terms of oral processing behavior or providing less familiar or “*warning*” sensations (i.e., bitterness, sourness, astringency, pungency) eliciting products, would be even larger with a truly neophobic population (e.g., FNS ≥ 55).

To conclude, the present study falls into the research stream acknowledging FN as a behavioral trait leading individuals to be more sensitive to sensory information in their environment. Among our main outcomes, we observed individuals with highest neophobic tendencies showing lower global olfactory performances. Moreover, we confirmed that selective/restrictive feeding attitudes result in a lower willingness to explore the chemosensory environment. Interestingly, we also observed a higher olfactory sensitiveness in individuals with medium neophobic traits compared to neophilics, and speculated that this effect may be mediated by a higher anxiety proneness. Individuals with neophobic traits also showed a lower extent of retronasal aroma release compared to more neophilic individuals, likely due to a different way they faced the oro-sensory contact with the sample chosen for this experiment. Thus, we can hypothesize that neophobics have developed an adaptive behaviour that protect them from an extended exposure with chemosensory stimuli, which might result in a more pleasant sensory experience less influenced by their higher arousal or anxiety state.

Since FN may lead to poorer dietary choices and nutritional deficiencies, based on the reported results we advocate the usage of anxiety-reducing treatment based on desensitization, as a suitable method to reduce these traits in adults with avoidance/restrictive tendencies^7^. As a final remark, this study supports the idea that neophobics’ feeding behavior are not be driven by a higher sensitivity to sensory stimuli but rather by higher levels of arousal toward foods that would lead them to limit their degree of exposure to the chemosensory world, which is crucial to guarantee the acceptance of a larger variety of food in the diet.

## Methods

### Participants

The present study is part of a broader large-scale investigation aiming to evaluate the Italian olfactory function according to biological, psychological, attitudinal and cognitive variables and to analyse the prevalence of olfactory disorders in Italy and its risk factors^52^.

We collected data from 83 healthy volunteers (aged 22–68 years old). Participants were recruited from the consumer database of the sensory laboratory of Edmund Mach Foundation through institutional mailing and social network (Facebook) announcements. Pregnant or lactating women, smokers, people with medical conditions or treatments that could modify olfactory functions e.g.^53^ were not included in the study. Informed, written consent according to the European Data Protection Regulation (UE 679/2016) was obtained from all participants. The present study was performed according to the principles established by the Declaration of Helsinki. The questionnaires and the olfactory protocol (i.e. the Sniffin’ Sticks procedure) were approved by the Institutional Ethics Committee of the University of Cagliari (the affiliation of one of the participating research groups)^52^. The NS protocol, not originally part of the broader study design^52^, was separately submitted to a local Ethics Committee, which provided an official waiver stating that its approval was not needed.

### Measurements

As part of the broader data collection proposed by Masala et al*.*^52^, interested and eligible respondents were given general information about the aim and the workflow of the study and were asked to fill out an online questionnaire collecting socio-demographic (gender, age) and the self-reported weight (kg) and height (m), later used to calculate the Body Mass Index (BMI) in kg/m^2^. As additional task from the original procedure described by Masala et al*.*^52^, the FNS and the STAI-T questionnaires were also collected remotely. All the respondents were asked to complete the online questionnaires prior to the start of the lab session. Later, participants were invited to the sensory laboratory of Edmund Mach Foundation where measures of olfactory functions and monitoring of retronasal aroma release were performed in a single session lasting approximately 90 min. During the lab session, volunteers were firstly given instructions on the olfactory assessment before starting the evaluation. Once the olfactory assessment was concluded, an interval of 10 min was enforced, during which participants were introduced to the retronasal aroma release protocol. Participants had been instructed to refrain from smoking, eating, drinking and brushing their teeth for at least 3 h prior the start of the lab session. Below, we briefly list all the measurements used for this study.

#### The Italian Food Neophobia Scale (FNS)

FN was measured using an Italian version of the common Food Neophobia Scale (FNS)^1^ developed and validated by Laureati et al*.*^8^. The FNS consists of 10 items each measured on a 7-point scale ranging from 1 *‘strongly disagree*’ to 7 ‘*strongly agree*’. The scores of five items reflecting neophilic food attitudes were reversed before analyses (see Supplementary Table S3). A FNS score, ranged theoretically from 10 to 70, was then computed for each individual as the sum of the scores given to the ten items, with higher scores resulting in higher neophobics tendencies. Cronbach’s α of the measure used was 0.88, nearly identical to the one reported by Laureati et al*.*^8^ (Cronbach’s α = 0.87).

#### The Italian Trait Anxiety Inventory Questionnaire (STAI-T)

Trait anxiety was measured through a validated Italian version of the trait subscale from the State-Trait Anxiety Inventory Questionnaire^33,34^ designed to measure relatively stable aspects of “anxiety proneness” (Cronbach’s α = 0.93). The STAI-T subscale consists of 20 statements, of which nine *anxiety-absent* and eleven *anxiety-present* items each measured on 4-point likert scale ranging from 1 “*almost never*” to 4 “*almost always*”. The scores from the nine anxiety*-*absent items were reversed before analyses (see Supplementary Table S4). The STAI-T score, ranged theoretically from 20 to 80, was then obtained by the sum of the scores from the 20 statements with higher scores reflecting higher levels of anxiety proneness^34^. STAI-T scores are commonly classified as “no or low anxiety” (20–37), “moderate anxiety” (38–44), and “high anxiety” (45–80)^34^.

#### The SNIFFIN’ sticks test

The Sniffin’ Sticks battery (Burghart, Wedel, Germany), developed by Hummel et al.^31^, was applied as psychophysical method to assess the olfactory function of our cohort. It comprises 3 subtests (OT = odor threshold; OD = odor discrimination; OI = odor identification) yielding 4 scores: T th,reshold score; D, discrimination score; I, identification score and TDI cumulative olfactory score^31^. The olfactory stimuli were presented using penlike odor dispensing devices placed in front of either nostril of participants for ~ 3 s. Participants were blindfolded in the OT and OD task. An interval of 5 min was enforced between each subtest.

##### Odor threshold test (OT)

The odor thresholds were obtained with triplets of odorant pens, each containing one *N*-butanol-impregnated pen and two odorless blanks, presented in 16 successive 1:2 dilution steps starting from a 4% solution using a single staircase method employing a triple alternative forced choice paradigm. The correct identification of two consecutive trials triggered a reversal of the staircase, while a wrong answer let the experimenter presenting the step-higher dilution triplet. The OT score, ranged theoretically from 1 to 16, was computed by the geometric mean of the last four staircase reversal points out a total of seven.

##### Odor discrimination test (OD)

In the OD test, participants were asked to sniff 16 triplets of odorant pens, each containing two identical odorant pens and a third with a different (target) odor. Odors were selected to be easily discriminated (more than 75% of times) by healthy individuals (for the name of odors, see^31^). In this forced choice paradigm, participants were asked to identify the “target” odor. One point was assigned for a correct answer while 0 for a wrong one. The OD score, ranged theoretically from 0 to 16, was obtained by computing the sum of the correct responses.

##### Odor identification test (OI)

The OI test was performed using 16 felt pens containing common odorants (for the name of odors see^31^) where participants were asked to identify the odor from a list of four alternative worded pictures, in a forced choice paradigm. As in the OD test, a correct answer was evaluated as 1 point, while an incorrect one as 0 point which resulted in a OI score, ranged theoretically from 0 to 16, computed by the sum of the correct responses.

##### TDI score

The cumulative TDI score represented the sum of the OT, OD and OI scores, ranging theoretically from 1 to 48. This score can be considered as a measure of the individuals’ overall olfactory function^31,35^ to be used to clinically arrange individuals as normosmic (i.e., normal olfactory function; TDI ≥ 30.75), hyposmic (impaired olfactory function; TDI ≤ 30.75) or functional anosmic (residual or absent olfactory function; TDI ≤ 16)^35^.

#### In vivo retronasal aroma release by SIFT-MS

##### Sample

A commercial strawberry flavored candy (Fruittella Caramelle Gelee; Perfetti Van Melle; Italy) was chosen as reference food matrix for our purposes according to the following criteria: (a) being widely common in Italy to avoid potentially refusals by neophobics; (b) having a flavoring composition easy to trace with SIFT-MS; (c) being a reproducible and easy to manage product.

##### SIFT-MS conditions

A commercial SIFT-MS (SYFT VOICE 200 ultra, Syft Ltd, New Zealand) was used to analyze in vivo the volatile organic compounds (VOCs) of the candy from the nose space of each participant. Exhaled air from participants’ nostrils was sampled through an ergonomic glass nosepiece with a silicone rubber tube. The nosepiece was connected to SIFT-MS with a PEEK tube (at room temperature for 30 cm and then heated at 110 °C). The instrument was calibrated daily with a mixture of standard gases (benzene, ethylene, isobutane, octafluorotoluene, hexaflurotoluene, toluene, p‐xylene, and 1,2,3,4‐tetrafluorobenzene; SRA Instruments S.p.A). The SIFT-MS was operated in SIM mode scanning 33 nominal *m*/*z* values related to eight selected VOCs.

We monitored seven different VOC’s, which represented the main aroma compounds present in the candy (2 alcohols, 4 esters and a carboxylic acid; ethyl maltol, 3-hexen-1-ol, ethyl 2-methylbutanoate, (Z)-3-hexenyl acetate, ethyl butanoate, ethyl hexanoate, 2-methylbutanoic acid) plus acetone as a marker of participants’ breathing cycle^54^. Acetone is naturally present in human breath and its released intensity is not affected during mastication^54^. Hence, it can be used to monitor participants’ breath rhythm during a nose-space task. Chemical ionization was achieved using H_3_O^+^, NO^+^, O_2_^+^ precursor ions and the overall scan time was set at 1 s. Data acquisition and processing was performed in LabSyft (Syft Technologies Ltd, version 1.6.2). A complete overview on SIFT-MS technique and on its benefits for real time analyses of odorants can be found in Langford et al*.*^55^.

##### Nose-Space analysis

A Nose-Space analysis (NS) through SIFT-MS was implemented to monitor in vivo retronasal aroma release. In brief, the task was carried out under a fixed-rhythm chewing (60 bpm) protocol supported by a video tool designed to train and help participants in following the procedure. The video showed an experimenter’s mouth chewing the strawberry flavored candy following a metronome that marked the tempo.

Prior to the start of the analysis, laboratory air (environmental background) was sampled for 20 s without any interaction with participants. Once the glass nosepiece had been fitted by the experimenter, participants were asked to breathe normally for 30 s to sample participants’ breath. Later, participants were asked to watch the video tool while simulating a real chewing phase to get familiar with the entire protocol. They were then instructed to put the jelly candy in the mouth and to press a button on the screen to let the experimenters know when the sample has been put in-mouth. The same instruction was given to alert the experimenter once the participant swallowed the sample. After swallowing the sample, participants had to continue breathing for 90 s, keeping the mouth closed. In total, the NS measurement lasted around 4 min. Taking into account the inter-individual variability on flavor release, e.g.^44–48^, at least three replicates per participant were performed to get a robust database. Between each replicate, participants were asked to rinse their mouth with mineral water, to have some unsalted bread, to rinse their mouth with mineral water again, and finally to wait at least 15 min before the next trial.

The entire procedure was conducted in an individual computerized sensory booth^56^ under cold white light located in a room with filtered air at constant temperature. FIZZ v2.50 (Biosystemes, Couternon, France) was used to guide participants along the entire protocol.

#### Statistical analysis

Variables of interest were summarized using means ± SD for normally distributed variables or median ± IQR for variables that did not fit normality assumptions, tested using Shapiro and Levene tests. For descriptive purposes, associations between demographic factors (i.e. age, BMI) and the variables of interest (FNS, STAI-T, olfactory performances and extent of retronasal aroma release) were assessed through Pearson or Spearman rank coefficients correlations, while differences in terms of gender were assessed through Welch Two Sample t-test or Mann–Whitney U test.

Participants were grouped according to their FN level using *cut-offs* proposed by^8^ and separate 1-way ANOVAs were performed for age and BMI to determinate whether a between group effect of such variables occurs as a function of FN level. Similarly, a *χ*^2^ test was used to evaluate differences on gender proportions between FN groups. These analyses were carried out to evaluate the potential confounding effect of variables whose influence on both olfactory performances and retronasal aroma release is known^31,35,37,47,48^. Then, to assess whether FN levels were associated to anxiety proneness, STAI-T scores were submitted to a 1-*way* ANOVA with FN level as “between subject factor”.

To assess differences between FN levels in olfactory performances, both single scores (OT, OD, OI) and the cumulative one (TDI) were submitted to separate Kruskall–Wallis tests with FN level as “between subject factor”. Dunn's test with Bonferroni correction was used as post hoc test when statistically significant differences between FN levels were observed.

For instrumental analyses, a timeslot of 2 min starting from the moment when participants put the sample in-mouth was considered for data analysis. Six parameters commonly used to analyze time-intensity (T-I) curves were extracted: the area under the curve (AUC), the maximum (I_max_), the median (I_median_), the average of the last five seconds of the NS session (I_end_), the time to reach the maximum (TI_max_) and the slope of the first descending section of the curve (Slope), assuming a linear relationship between time and the logarithm of peak intensity^57,58^. Moreover, duration of oral processing was calculated for each replicate by subtracting the time when participants put the sample in-mouth to the time they swallowed it. Lastly, participants’ breathing rate was estimated for each replicate by counting the local minima sites from the acetone curves within the task.

After data pre-processing and treatments following the guidelines suggested by^57,58^, a dataset with 262 rows (participants*replicates) and 42 columns, organized in 6 groups (SIFT-MS parameters; AUC, I_max_, I_median_, I_end_, TI_max_, Slope) of 7 variables each (monitored VOCs), was built and then submitted to a Multiple Factor Analysis (MFA) to visualize global differences between FN levels in terms of aroma release. Each group of variables was log-transformed and scaled to unit of variance prior the analysis. The holistic exploration was followed by a univariate approach where all the parameters investigated plus the duration of oral processing and the breathing rate were analyzed with separate Kruskall Wallis tests with FN level as “between subject factor”. Dunn’s test with Bonferroni correction was used as post hoc test whenever a statistically significant difference between FN levels was observed.

All data analyses were run in R software v3.6.3^59^. All tests were two-tailed, a *p* < 0.05 was considered as significant while the range 0.05 ≤ *p* ≤ 0.10 was accepted as a trend.

## Supplementary information

Supplementary Information.

## Data Availability

The datasets generated during and/or analyzed during the current study are available from the corresponding author on reasonable request.

## References

[1] Pliner P, Hobden K (1992). Development of a scale to measure the trait of food neophobia in humans. Appetite.

[2] Rozin P (1976). The Selection of Foods by Rats, Humans, and Other Animals. Adv. Study Behav..

[3] Fischler C (1990). L'homme omnivore.

[4] Rozin P, Vollmecke TA (1986). Food likes and dislikes. Annu. Rev. Nutr..

[5] Dovey TM, Staples PA, Gibson EL, Halford JCG (2008). Food neophobia and ‘picky/fussy’ eating in children: a review. Appetite.

[6] Birch LL (1998). Development of food acceptance patterns in the first years of life. Proc. Nutr. Soc..

[7] Marcontell DK, Laster AE, Johnson J (2002). Cognitive-behavioral treatment of food neophobia in adults. J. Anxiety Disord..

[8] Laureati M (2018). Associations between food neophobia and responsiveness to “warning” chemosensory sensations in food products in a large population sample. Food Qual. Prefer..

[9] Jaeger SR, Rasmussen MA, Prescott J (2017). Relationships between food neophobia and food intake and preferences: findings from a sample of New Zealand adults. Appetite.

[10] Demattè ML, Endrizzi I, Gasperi F (2014). Food neophobia and its relation with olfaction. Front. Psychol..

[11] Knaapila A (2011). Food neophobia in young adults: Genetic architecture and relation to personality, pleasantness and use frequency of foods, and body mass index-A twin study. Behav. Genet..

[12] Tuorila H, Lähteenmäki L, Pohjalainen L, Lotti L (2001). Food neophobia among the Finns and related responses to familiar and unfamiliar foods. Food Qual. Prefer..

[13] Raudenbush B, Schroth F, Reilley S, Frank RA (1998). Food neophobia, odor evaluation and exploratory sniffing behavior. Appetite.

[14] Raudenbush B, Corley N, Flower NR, Kozlowski A, Meyer B (2003). Cephalic phase salivary response differences characterize level of food neophobia. Appetite.

[15] Proserpio C, Laureati M, Invitti C, Pagliarini E (2018). Reduced taste responsiveness and increased food neophobia characterize obese adults. Food Qual. Prefer..

[16] Knaapila AJ (2015). Food neophobia associates with lower dietary quality and higher BMI in Finnish adults. Public Health Nutr..

[17] Knaapila A (2007). Food neophobia shows heritable variation in humans. Physiol. Behav..

[18] Alley, T. R. Conceptualization and measurement of human food neophobia, in *Food Neophobia: Behavioral and Biological Influences* (2018). doi:10.1016/B978-0-08-101931-3.00009-4.

[19] Monteleone E (2017). Exploring influences on food choice in a large population sample: The Italian Taste project. Food Qual. Prefer..

[20] Spinelli S (2018). Personality traits and gender influence liking and choice of food pungency. Food Qual. Prefer..

[21] Meiselman HL, King SC, Gillette M (2010). The demographics of neophobia in a large commercial US sample. Food Qual. Prefer..

[22] Hummel T, Nordin S (2005). Olfactory disorders and their consequences for quality of life. Acta Otolaryngol..

[23] Prescott J (1999). Flavour as a psychological construct: implications for perceiving and measuring the sensory qualities of foods. Food Qual. Prefer..

[24] Small DM, Jones-Gotman M, Zatorre RJ, Petrides M, Evans AC (1997). Flavor processing: more than the sum of its parts. NeuroReport.

[25] Prescott J, Burns J, Frank RA (2010). Influence of odor hedonics, food-relatedness, and motivational state on human sniffing. Chemosens. Percept..

[26] Demattè ML (2013). Food neophobia and its relation with olfactory ability in common odour identification. Appetite.

[27] Monnery-Patris S (2015). Smell differential reactivity, but not taste differential reactivity, is related to food neophobia in toddlers. Appetite.

[28] Farrow CV, Coulthard H (2012). Relationships between sensory sensitivity, anxiety and selective eating in children. Appetite.

[29] Raudenbush B, Capiola A (2012). Physiological responses of food neophobics and food neophilics to food and non-food stimuli. Appetite.

[30] Pliner P, Melo N (1997). Food neophobia in humans: Effects of manipulated arousal and individual differences in sensation seeking. Physiol. Behav..

[31] Hummel T, Sekinger B, Wolf SR, Pauli E, Kobal G (1997). ‘Sniffin’ Sticks’: olfactory performance assessed by the combined testing of odor identification, odor discrimination and olfactory threshold. Chem. Senses.

[32] Smith D, Španěl P (2005). Selected ion flow tube mass spectrometry (SIFT-MS) for on-line trace gas analysis. Mass Spectrom. Rev..

[33] Spielberger, C., Gorsuch, R., Lushene, R., Vagg, P. R. & Jacobs, G. *Manual for the State-Trait Anxiety Inventory (Form Y1–Y2)*, vol. 4 (Consulting Psychologists Press, Palo Alto) (1983).

[34] Pedrabissi, L. & Santinello, M. Verifica della validità dello STAI forma Y di Spielberger [Verification of the validity of the STAI, Form Y, by Spielberger]. *Giunti Organ. Spec.* **191–192**, 11–14 (1989).

[35] Oleszkiewicz A, Schriever VA, Croy I, Hähner A, Hummel T (2019). Updated Sniffin’ Sticks normative data based on an extended sample of 9139 subjects. Eur. Arch. Oto-Rhino-Laryngology.

[36] Hummel T, Kobal G, Gudziol H, Mackay-Sim A (2007). Normative data for the “Sniffin’ Sticks” including tests of odor identification, odor discrimination, and olfactory thresholds: an upgrade based on a group of more than 3,000 subjects. Eur. Arch. Oto-Rhino-Laryngol..

[37] Schöpf V, Kollndorfer K, Pollak M, Mueller CA, Freiherr J (2015). Intranasal insulin influences the olfactory performance of patients with smell loss, dependent on the body mass index: a pilot study. Rhinology.

[38] Besser G (2020). Body-mass-index associated differences in ortho- and retronasal olfactory function and the individual significance of olfaction in health and disease. J. Clin. Med..

[39] Kassambara, A. *Multivariate analysis II: Practical Guide To Principal Component Methods in R: PCA, M (CA), FAMD, MFA, HCPC, factoextra*. Sthda (2017).

[40] Roessner V, Bleich S, Banaschewski T, Rothenberger A (2005). Olfactory deficits in anorexia nervosa. Eur. Arch. Psychiatry Clin. Neurosci..

[41] Gottfried JA, Dolan RJ (2003). The nose smells what the eye sees: Crossmodal visual facilitation of human olfactory perception. Neuron.

[42] Cain WS (1979). To know with the nose: keys to odor identification. Science (80–)..

[43] Paulus MP, Stein MB (2006). An insular view of anxiety. Biol. Psychiatr..

[44] Repoux M, Sémon E, Feron G, Guichard E, Labouré H (2012). Inter-individual variability in aroma release during sweet mint consumption. Flavour Fragr. J..

[45] Labouré H, Repoux M, Courcoux P, Feron G, Guichard E (2014). Inter-individual retronasal aroma release variability during cheese consumption: role of food oral processing. Food Res. Int..

[46] Pedrotti M, Spaccasassi A, Biasioli F, Fogliano V (2019). Ethnicity, gender and physiological parameters: their effect on in vivo flavour release and perception during chewing gum consumption. Food Res. Int..

[47] Ruijschop RMAJ, Burgering MJM, Jacobs MA, Boelrijk AEM (2009). Retro-nasal aroma release depends on both subject and product differences: a link to food intake regulation?. Chem. Senses.

[48] Ruijschop RMAJ (2009). Effects of bite size and duration of oral processing on retro-nasal aroma release—features contributing to meal termination. Br. J. Nutr..

[49] Paulus MP (2013). The breathing conundrum—interoceptive sensitivity and anxiety. Depress. Anxiety.

[50] Masaoka Y, Homma I (2001). The effect of anticipatory anxiety on breathing and metabolism in humans. Respir. Physiol..

[51] Paulus MP, Stein MB (2010). Interoception in anxiety and depression. Brain Struct. Funct..

[52] Masala C (2019). An Italian population-based study of the prevalence of olfactory impairment. Chem. Senses.

[53] Beecher K, St John J, Chehrehasa F (2018). Factors that modulate olfactory dysfunction. Neural Regener. Res..

[54] Biasioli F, Gasperi F, Yeretzian C, Märk TD (2011). PTR-MS monitoring of VOCs and BVOCs in food science and technology. TrAC Trends Anal. Chem..

[55] Langford VS, Padayachee D, McEwan MJ, Barringer SA (2019). Comprehensive odorant analysis for on-line applications using selected ion flow tube mass spectrometry (SIFT-MS). Flavour Fragr. J..

[56] ISO. Sensory analysis-General guidance for the design of test rooms. *ISO Standard 8589* (2007).

[57] Normand V, Avison S, Parker A (2004). Modeling the kinetics of flavour release during drinking. Chem. Senses.

[58] Charles M (2015). Understanding flavour perception of espresso coffee by the combination of a dynamic sensory method and in-vivo nosespace analysis. Food Res. Int..

[59] R Core Team. *R: A Language and Environment for Statistical Computing* (Vienna, 2019). https://www.R-project.org/. Accessed 15 Oct 2020.

