# Supplementary material for: Arousal influences olfactory abilities in adults with different degree of food neophobia
Source: Sci Rep. 2020 Nov 25;10:20538. doi: 10.1038/s41598-020-77428-w (PMC7689524; doi:10.1038/s41598-020-77428-w)
Supplement: Supplementary file 1 — Supplementary Information. [file 41598_2020_77428_MOESM1_ESM.pdf]

Arousal influences olfactory abilities in adults with different degree of food neophobia

Leonardo Menghi[1,2,3], Iuliia Khomenko[3], Michele Pedrotti[3,4], Danny Cliceri[3], Eugenio Aprea[1,3], Isabella Endrizzi[3], Annachiara Cavazzana[5], Franco Biasioli[3], Davide Giacalone[2] & **Flavia Gasperi[1,3*]**

[1]University of Trento - Center Agriculture Food Environment; Via E. Mach, 1 – San Michele all'Adige (TN) – 38010 – Italy;

[2]University of Southern Denmark – Department of Technology and Innovation; Campusvej 55 – Odense – 5230 – Denmark;

[3]Department of Food Quality and Nutrition, Research and Innovation Centre, Fondazione Edmund Mach; Via E. Mach, 1 – San Michele all'Adige (TN) – 38010 – Italy;

[4]Wageningen University – Department of Food Quality and Design, P.O. Box 8129, 6700 EV Wageningen, The Netherlands;

[5]Technische Universität Dresden - Department of Otorhinolaryngology, Smell and Taste Clinic; Fetscherstraße 74 - Dresden – 01307 – Germany.

*Correspondence to: Flavia Gasperi
E-mail: flavia.gasperi@unitn.it

SUPPLEMENTARY MATERIALS

Participants (n=83)		Normative Data [1]							
Age group	TDI (Mean ± SD)	5th	10th	25th	50th	75th	90th	95th	SD
21-30 (n=22)	36.65 ± 4.04	29.50	30.75	33.06	**35.75**	**38.50**	41.50	43.09	4.20
31-40 (n=17)	36.04 ± 6.53	28.74	30.50	33.00	**35.50**	**38.50**	40.50	42.01	4.03
41-50 (n=20)	34.62 ± 3.23	25.50	28.15	**31.50**	**34.75**	37.00	39.50	41.00	4.73
51-60 (n=20)	34.26 ± 3.7	25.33	27.25	30.34	**33.00**	**36.25**	38.50	40.18	4.69
61-70 (n=4)	36.12 ± 3.83	22.50	24.88	28.50	31.63	**34.25**	**36.50**	38.25	4.78

Table S1: TDI average scores are presented separately for the age groups suggested by normative data [1] comprising our cohort (Participants). Normative data are listed as mean values within age groups-related percentiles ± SD. In bold the age group-related range of performance in which individuals felt in comparison with normative values.

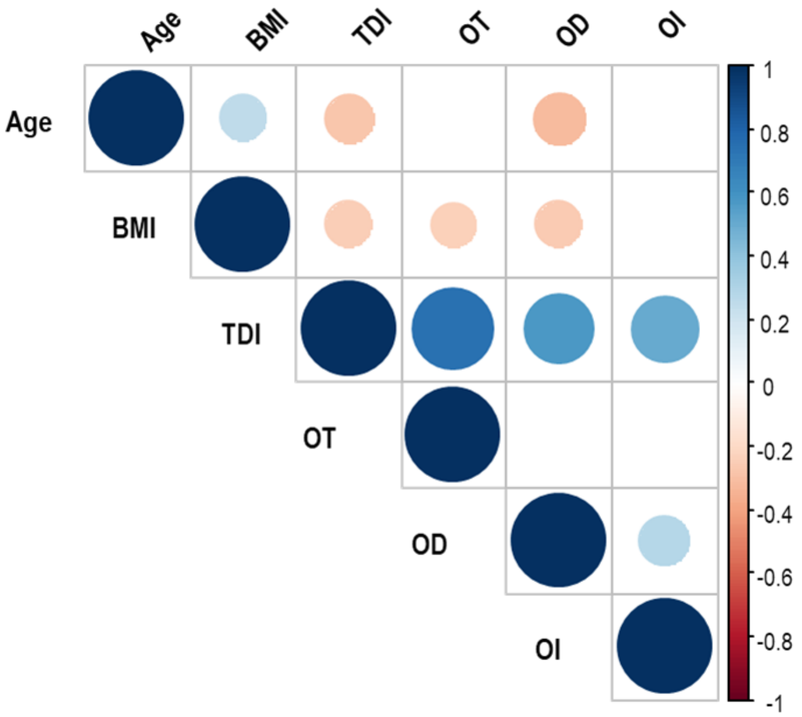

Figure S1: Correlations between olfactory performance scores, age and BMI. Only statistically significant differences ($p<0.05$) according to Spearman's rank correlation coefficients are depicted. Abbreviation: TDI = Odor Threshold, Discrimination, Identification composite score; OT= Odor Threshold; OD= Odor Discrimination; OI= Odor Identification.

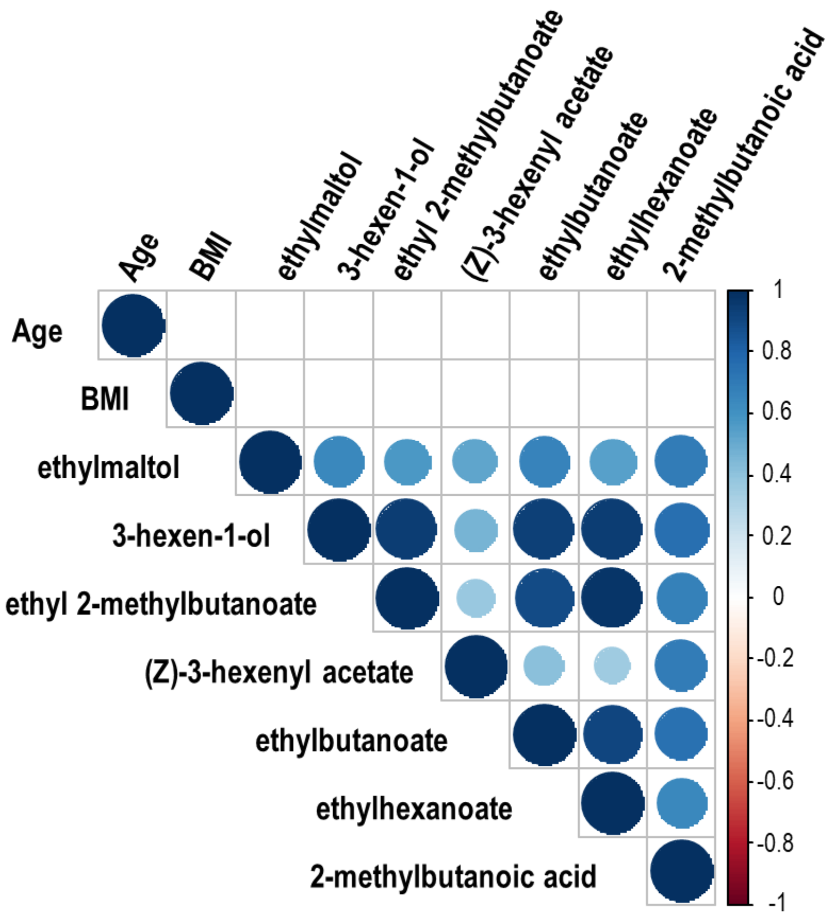

Figure S2: Correlations between areas under the curves from the 7 monitored compounds, age and BMI. Only statistically significant differences ($p<0.05$) according to Spearman's rank correlation coefficients are depicted.

Sum formula	Variable	F (n=48)	M (n=35)	U	*p-value*
/	TDI	35.87 ± 5.00	35.25 ± 5.62	809.50	0.78
/	OT	10.75 ± 3.31	10 ± 4.75	716	0.25
/	OD	12 ± 2.00	13 ± 2.50	695	0.17
/	OI	13 ± 2.00	13 ± 2.00	832.50	0.94
$C_7H_8O_3$	ethylmaltol	3.32 ± 0.33	3.29 ± 0.28	775	0.55
$C_6H_{12}O$	3-hexen-1-ol	8.83 ± 0.39	8.82 ± 0.60	802	0.72
$C_7H_{14}O_2$	ethyl 2-methylbutanoate	7.04 ± 0.63	6.96 ± 0.89	779	0.57
$C_8H_{14}O_2$	(Z)-3-hexenyl acetate	7.51 ± 0.5	7.43 ± 0.49	701	0.19
$C_6H_{12}O_2$	ethylbutanoate	9.14 ± 0.37	9.09 ± 0.61	772	0.53
$C_8H_{16}O_2$	ethylhexanoate	9.09 ± 0.57	9.17 ± 0.87	797	0.69
$C_5H_{10}O_2$	2-methylbutanoic acid	6.91 ± 0.27	6.82 ± 0.35	689	0.16

Table S2: Differences on both olfactory performances and the extent of retronasal aroma release (area under the curve) for all the 7 monitored compounds as a function of gender (F= Females; M= Males) according to Mann Whitney-U test. Values are listed as median values within gender ± IQR. Abbreviation: TDI = Odor Threshold, Discrimination, Identification composite score; OT= Odor Threshold; OD= Odor Discrimination; OI= Odor Identification.

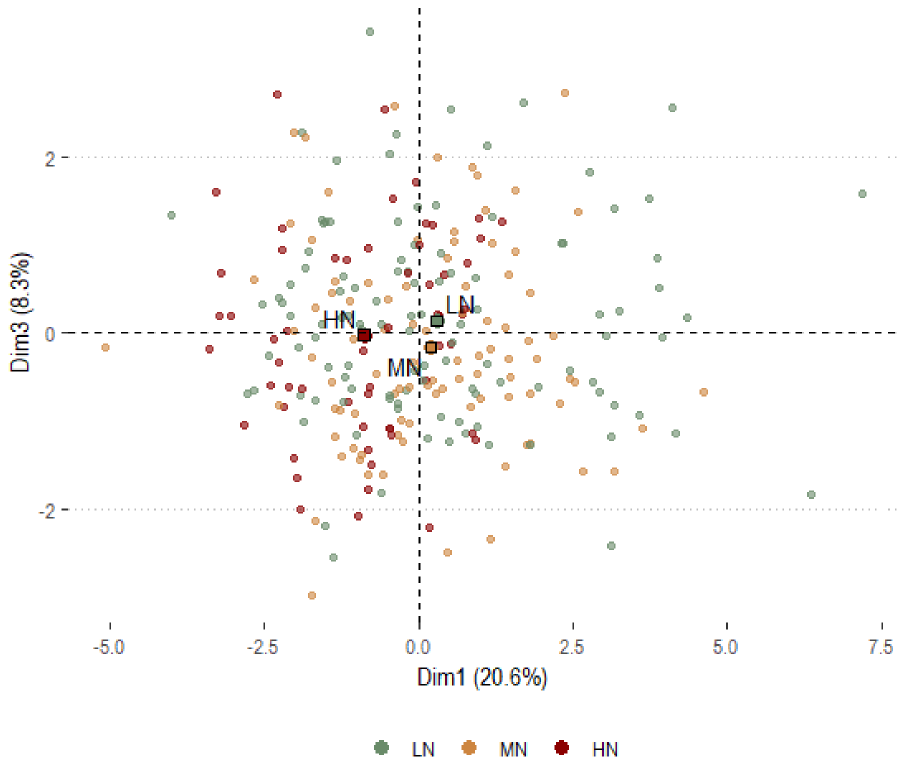

Figure S3: First and third principal components of the individual factor map from the MFA model based on matrices of SIFT-MS parameters. Large squares (■ ■ ■) are MFA centroids while the circles (● ● ●) represent participants' position in the bi-dimensional space colored according to their FN level (**LN**, **MN**, **HN**).

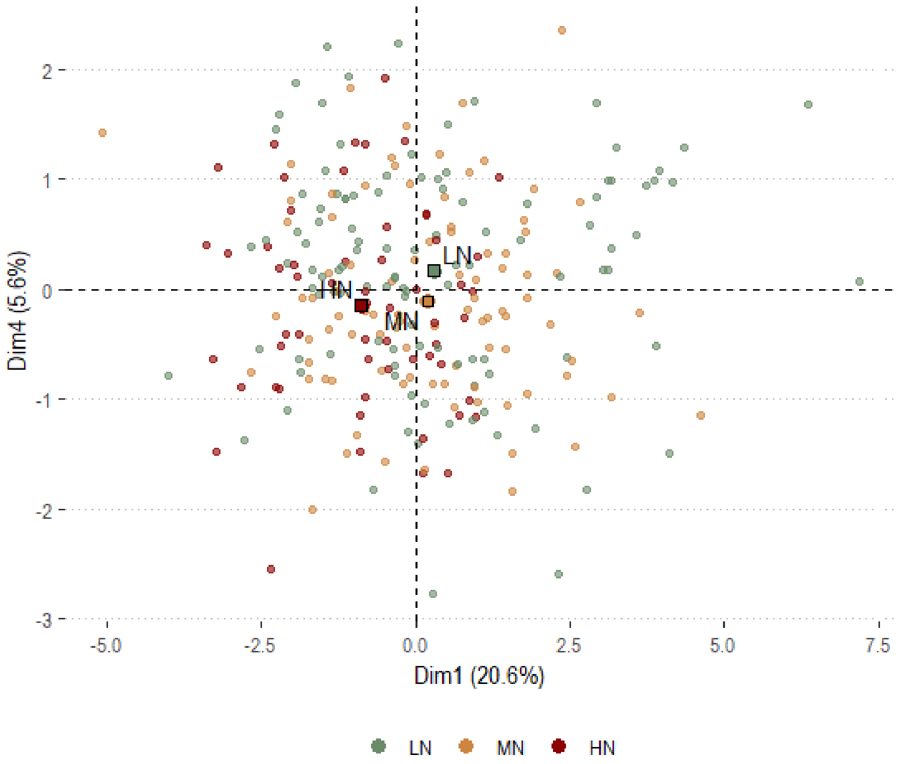

Figure S4: First and fourth principal components of the individual factor map from the MFA model based on matrices of SIFT-MS parameters. Large squares (■ ■ ■) are MFA centroids while the circles (● ● ●) represent participants' position in the bi-dimensional space colored according to their FN level (**LN**, **MN**, **HN**).

Food Neophobia Scale – Original version [2]	Italian Version of the Food Neophobia Scale [3]
1. I am constantly sampling new and different foods **(R)**	1. Provo continuamente cibi nuovi e differenti dal solito **(R)**
2. I don't trust new foods	2. Nella scelta del cibo non mi fido delle novità
3. If I don't know what is in a food, I won't try it	3. Se non conosco un alimento, non lo assaggio
4. I like foods from different countries **(R)**	4. Mi piace il cibo di diversi Paesi **(R)**
5. Ethnic food looks too weird to eat	5. Il cibo etnico mi sembra molto strano per poterlo mangiare
6. At dinner parties, I will try a new food **(R)**	6. Alle cene con amici mi piace assaggiare cibi nuovi **(R)**
7. I am afraid to eat things I have never had before	7. Ho timore a mangiare cibi mai assaggiati
8. I am very particular about the foods I will eat	8. Sono schizzinoso riguardo al cibo che mangio
9. I will eat almost anything **(R)**	9. Generalmente mangio quasi tutto **(R)**
10. I like to try new ethnic restaurants **(R)**	10. Mi piace provare nuovi ristoranti etnici **(R)**

Table S3: Original (left side of the table) [2] and Italian (right side of the table) [3] version of the Food Neophobia Scale (FNS). **(R)** indicate the items of FNS reflecting neophilic food attitudes that have to be reversed before analyses.

The Trait Anxiety Inventory Questionnaire – Original version [4]	Italian Version of the Trait Anxiety Inventory Questionnaire [5]
1. I feel pleasant **(R)**	1. Mi sento bene **(R)**
2. I feel nervous and restless	2. Mi sento teso/a e irrequieto/a
3. I feel satisfied with myself **(R)**	3. Sono soddisfatto/a di me stesso/a **(R)**
4. I wish I could be as happy as others seem to be	4. Vorrei poter essere felice come sembrano essere gli altri
5. I feel like a failure	5. Mi sento un/una fallito/a
6. I feel rested **(R)**	6. Mi sento riposato/a **(R)**
7. I am "calm, cool, and collected" **(R)**	7. Io sono calmo/a, tranquillo/a e padrone/a di me **(R)**
8. I feel that difficulties are piling up so that I cannot overcome them	8. Sento che le difficoltà si accumulano tanto da non poterle superare
9. I worry too much over something that really doesn't matter	9. Mi preoccupo troppo di cose che in realtà non hanno importanza
10. I am happy **(R)**	10. Sono felice **(R)**
11. I have disturbing thoughts	11. Mi vengono pensieri negativi
12. I lack self-confidence	12. Manco di fiducia in me stesso
13. I feel secure **(R)**	13. Mi sento sicuro **(R)**
14. I make decisions easily **(R)**	14. Prendo decisioni facilmente **(R)**
15. I feel inadeguate	15. Mi sento inadeguato
16. I am content **(R)**	16. Sono contento **(R)**
17. Some unimportant thought runs through my mind and bothers me	17. Pensieri di scarsa importanza mi passano per la mente e mi infastidiscono
18. I take disappointments so keenly that I can't put them out of my mind	18. Vivo le delusioni con tanta partecipazione da non poter togliermele dalla testa
19. I am a steady person **(R)**	19. Sono una persona costante **(R)**
20. I get in a state of tension or turmoil as I think over my recent concerns and interests	20. Divento teso e turbato quando penso alle mie attuali abitudini

Table S4: Original (left side of the table) [4] and Italian version (right side of the table) [5] of the Trait Anxiety Inventory Questionnaire (STAI-T). **(R)** indicate the items of the STAI-T reflecting *anxiety-absent* attitudes that have to be reversed before analyses.

REFERENCES

1. Oleszkiewicz, A., Schriever, V. A., Croy, I., Hähner, A. & Hummel, T. Updated Sniffin' Sticks normative data based on an extended sample of 9139 subjects. *Eur. Arch. Oto-Rhino-Laryngology* **276**, 719–728 (2019).

2. Pliner, P. & Hobden, K. Development of a scale to measure the trait of food neophobia in humans. *Appetite* (1992) doi:10.1016/0195-6663(92)90014-W.

3. Laureati, M. *et al.* Associations between food neophobia and responsiveness to "warning" chemosensory sensations in food products in a large population sample. *Food Qual. Prefer.* (2018) doi:10.1016/j.foodqual.2018.02.007.

4. Spielberger, C., Gorsuch, R., Lushene, R., Vagg, P. R. & Jacobs, G. *Manual for the State-Trait Anxiety Inventory (Form Y1 – Y2)*. *Palo Alto, CA: Consulting Psychologists Press;* vol. IV (1983).

5. Pedrabissi, L. & Santinello, M. Verifica della validità dello STAI forma Y di Spielberger. [Verification of the validity of the STAI, Form Y, by Spielberger.]. *Giunti Organ. Spec.* (1989).
